# Supplementary material for: (−)-Gallocatechin Gallate: A Novel Chemical Marker to Distinguish Triadica cochinchinensis Honey
Source: Foods. 2024 Jun 14;13(12):1879. doi: 10.3390/foods13121879 (PMC11203108; doi:10.3390/foods13121879)
Supplement: Supplementary file 1 [file foods-13-01879-s001.zip › Supplementary Table S1.pdf]

Table S1

Standard information for 204 flavonoids.

No.	Compounds	Abbreviations	Classification
1	Astilbin	Flavonoid_01	Flavanonols
2	Miquelianin	Flavonoid_02	Flavonols
3	4,4-Dimethoxychalcone	Flavonoid_03	Chalcones
4	Isosilybin	Flavonoid_04	Flavanonols
5	7-Methoxyisoflavone	Flavonoid_05	Isoflavanones
6	Diosmetin	Flavonoid_06	Flavones
7	Troxerutin	Flavonoid_07	-
8	5-Methyl-7-methoxyisoflavone	Flavonoid_08	Isoflavanones
9	Genistein	Flavonoid_09	Isoflavanones
10	3,4-Dihydroxyflavone	Flavonoid_10	Flavones
11	Hydroxysafflor yellow A	Flavonoid_11	-
12	Liquiritigenin	Flavonoid_12	Flavanones
13	Isoorientin	Flavonoid_13	Flavone glycosides
14	Nobiletin	Flavonoid_14	Flavones
15	Neohesperidin	Flavonoid_15	Flavanones
16	7,4-Di-O-methylapigenin	Flavonoid_16	Flavones
17	Flavokawain C	Flavonoid_17	Phenonic acids
18	Baicalein	Flavonoid_18	Flavones
19	beta-Mangostin	Flavonoid_19	Xanthones
20	Sagittatoside A	Flavonoid_20	Flavonols
21	Tangeretin	Flavonoid_21	Flavones
22	Neohesperidin dihydrochalcone	Flavonoid_22	Chalcones
23	Quercetin	Flavonoid_23	Flavonols
24	4,5-Dihydroxyflavone	Flavonoid_24	Flavones
25	Apigenin-7-glucuronide	Flavonoid_25	Flavones
26	Formononetin	Flavonoid_26	Isoflavanones
27	Flavonol	Flavonoid_27	Flavonols
28	Scutellarin	Flavonoid_28	Flavones
29	IKarisoside A	Flavonoid_29	Flavones
30	Isoliquiritigenin	Flavonoid_30	Chalcones
31	Naringin Dihydrochalcone	Flavonoid_31	Chalcones
32	Daidzin	Flavonoid_32	Isoflavanones
33	Oroxin A	Flavonoid_33	Flavones
34	Isoginkgetin	Flavonoid_34	Biflavonoids
35	Syringaldehyde	Flavonoid_35	Phenonic acids
36	Icariside I	Flavonoid_36	Flavonols
37	Kurarinone	Flavonoid_37	-
38	Corylifol A	Flavonoid_38	Isoflavanones
39	Trimethylapigenin	Flavonoid_39	Flavones
40	Dihydromyricetin	Flavonoid_40	Flavanonols
41	Hesperetin	Flavonoid_41	Flavones

Continue for Table S1

No.	Compounds	Abbreviations	Classification
42	Nicotiflorin	Flavonoid_42	Flavones
43	Kaempferol	Flavonoid_43	Flavonols
44	4-Hydroxychalcone	Flavonoid_44	Chalcones
45	3,7,4-Trihydroxyflavone	Flavonoid_45	Flavonols
46	Eupatorin	Flavonoid_46	Flavones
47	Ginkgetin	Flavonoid_47	Biflavonoids
48	Narirutin	Flavonoid_48	Flavanones
49	Baicalin	Flavonoid_49	Flavones
50	Luteolin	Flavonoid_50	Flavones
51	3-Methoxypuerarin	Flavonoid_51	Flavone glycosides
52	Kaempferol 3-neohesperidoside	Flavonoid_52	Flavonols
53	Cardamonin	Flavonoid_53	Phenonic acids
54	Isorhamnetin-3-O-neohespeidoside	Flavonoid_54	Flavonols
55	(-)-Catechin	Flavonoid_55	Flavanols
56	Taxifolin	Flavonoid_56	Flavanonols
57	Rutin	Flavonoid_57	Flavonols
58	Isorhamnetin	Flavonoid_58	Flavonols
59	Hispidulin	Flavonoid_59	Flavones
60	Kushenol A	Flavonoid_60	Other flavonoids
61	Chrysin	Flavonoid_61	Flavones
62	Methylophiopogonanone B	Flavonoid_62	Phenonic acids
63	Tectorigenin	Flavonoid_63	Isoflavanones
64	Scutellarein	Flavonoid_64	Flavones
65	Phlorizin	Flavonoid_65	Chalcones
66	Puerarin	Flavonoid_66	Isoflavanones
67	Fisetin	Flavonoid_67	Flavonols
68	Mangiferin	Flavonoid_68	Xanthones
69	Liquiritin	Flavonoid_69	Flavanones
70	Licochalcone E	Flavonoid_70	Chalcones
71	Galangin	Flavonoid_71	Flavones
72	Apigenin	Flavonoid_72	Flavones
73	Genistin	Flavonoid_73	Isoflavanones
74	Schaftoside	Flavonoid_74	Flavone glycosides
75	Licochalcone C	Flavonoid_75	Chalcones
76	Sakuranetin	Flavonoid_76	Flavones
77	Epimedin A	Flavonoid_77	-
78	Eriocitrin	Flavonoid_78	Flavanones
79	Ligustroflavone	Flavonoid_79	Phenonic acids
80	(-)-Gallocatechin gallate	Flavonoid_80	Flavanols
81	Tricin	Flavonoid_81	Flavones
82	Chrysosplenetin	Flavonoid_82	Flavones
83	Epmedin C	Flavonoid_83	-

Continue for Table S1

No.	Compounds	Abbreviations	Classification
84	(-)-Epigallocatechin	Flavonoid_84	Flavanols
85	Methylnissolin-3-O-glucoside	Flavonoid_85	-
86	Avicularin	Flavonoid_86	Flavonols
87	Noricaritin	Flavonoid_87	Flavonols
88	5-O-Demethylnobiletin	Flavonoid_88	Flavones
89	Silibinin	Flavonoid_89	Flavanonols
90	Cynaroside	Flavonoid_90	Flavones
91	Morusin	Flavonoid_91	Other flavonoids
92	Myricetin	Flavonoid_92	Flavonols
93	Bavachin	Flavonoid_93	Flavanones
94	Epimedin B	Flavonoid_94	-
95	Calycosin	Flavonoid_95	Isoflavanones
96	Calycosin-7-O-β-D-glucoside	Flavonoid_96	Isoflavanones
97	Xanthohumol	Flavonoid_97	Chalcones
98	Hyperoside	Flavonoid_98	Flavonols
99	Acacetin	Flavonoid_99	Flavones
100	Sophoraflavanone G	Flavonoid_100	Flavanones
101	Hesperidin	Flavonoid_101	Flavanones
102	Theaflavin	Flavonoid_102	-
103	Scutellarein tetramethyl ether	Flavonoid_103	Flavones
104	Corylin	Flavonoid_104	Isoflavanones
105	(E)-Flavokawain A	Flavonoid_105	Phenonic acids
106	Typhaneoside	Flavonoid_106	Flavonols
107	Wogonin	Flavonoid_107	Flavones
108	Pinocembrin	Flavonoid_108	Flavanones
109	Deguelin	Flavonoid_109	-
110	Kaempferide	Flavonoid_110	Flavonols
111	Irisflorentin	Flavonoid_111	Isoflavanones
112	Echinatin	Flavonoid_112	Chalcones
113	Taxifolin 7-O-rhamnoside	Flavonoid_113	Flavanonols
114	6-O-Acetylglycitin	Flavonoid_114	Isoflavanones
115	Quercitrin	Flavonoid_115	Flavonols
116	4-O-Methylbavachalcone	Flavonoid_116	Chalcones
117	Engeletin	Flavonoid_117	Flavanonols
118	Narcissin	Flavonoid_118	Flavones
119	Astragalin	Flavonoid_119	Flavonols
120	Bavachinin	Flavonoid_120	Flavanones
121	Licoisoflavone A	Flavonoid_121	Isoflavanones
122	7-Hydroxy-4H-chromen	Flavonoid_122	Phenonic acids
123	2-O-Galloylhyperin	Flavonoid_123	Flavonols
124	Silychristin	Flavonoid_124	Flavanonols
125	Amentoflavone	Flavonoid_125	Biflavonoids

Continue for Table S1

No.	Compounds	Abbreviations	Classification
126	Eriodictyol	Flavonoid_126	Flavanones
127	Daidzein	Flavonoid_127	Isoflavanones
128	5-Hydroxyflavone	Flavonoid_128	Flavones
129	Mulberrin	Flavonoid_129	Other flavonoids
130	Isobavachalcone	Flavonoid_130	Chalcones
131	Neobavaisoflavone	Flavonoid_131	Isoflavanones
132	Alpinetin	Flavonoid_132	Flavanones
133	β-Anhydroicaritin	Flavonoid_133	-
134	Loureirin B	Flavonoid_134	Chalcones
135	Theaflavin 3-digallate	Flavonoid_135	-
136	6,2-Dihydroxyflavone	Flavonoid_136	Flavones
137	Tiliroside	Flavonoid_137	Flavonols
138	Baimaside	Flavonoid_138	Flavonols
139	Dihydrokaempferol	Flavonoid_139	Flavanonols
140	Licoflavone A	Flavonoid_140	Flavones
141	Isosakuranin	Flavonoid_141	Flavanones
142	Sciadopitysin	Flavonoid_142	-
143	Jaceosidin	Flavonoid_143	Flavones
144	Kavain	Flavonoid_144	Phenonic acids
145	Procyanidin B2	Flavonoid_145	Anthocyanins
146	Icariin	Flavonoid_146	Flavonols
147	(-)-Epicatechin	Flavonoid_147	Flavanols
148	Baohuoside I	Flavonoid_148	Flavonols
149	Farrerol	Flavonoid_149	Flavanones
150	(-)-Catechin gallate	Flavonoid_150	Flavanols
151	Sinensetin	Flavonoid_151	Flavones
152	Ononin	Flavonoid_152	Isoflavanones
153	Glycitin	Flavonoid_153	Isoflavanones
154	Vitexin	Flavonoid_154	Flavone glycosides
155	Spinosin	Flavonoid_155	Flavone glycosides
156	Wogonoside	Flavonoid_156	Flavones
157	Isomangiferin	Flavonoid_157	Xanthones
158	Isosakuranetin	Flavonoid_158	Flavanones
159	Icaritin	Flavonoid_159	Flavonols
160	Apigenin 7-glucoside	Flavonoid_160	Flavones
161	Diosmin	Flavonoid_161	Flavones
162	Tectochrysin	Flavonoid_162	Flavones
163	4-Hydroxychalcone	Flavonoid_163	Chalcones
164	5-Methoxyflavone	Flavonoid_164	Flavones
165	Homoplantaginin	Flavonoid_165	Flavones
166	Genkwanin	Flavonoid_166	Flavones
167	Oroxin B	Flavonoid_167	Flavones

Continue for Table S1

No.	Compounds	Abbreviations	Classification
168	5,7,3,4-Tetramethoxyflavone	Flavonoid_168	Flavones
169	Quercetin3-O-(6-galloyl)-β-D-galactopyranoside	Flavonoid_169	Flavonols
170	Licoflavonol	Flavonoid_170	Flavonols
171	6-Hydroxyflavone	Flavonoid_171	Flavones
172	Kaempferitrin	Flavonoid_172	Flavonols
173	6-Methylflavone	Flavonoid_173	Flavones
174	Glabridin	Flavonoid_174	Isoflavanones
175	Afzelin	Flavonoid_175	Flavonols
176	Naringenin-7-glucoside	Flavonoid_176	Flavanones
177	Phloretin	Flavonoid_177	Chalcones
178	Myricitrin	Flavonoid_178	Flavonols
179	Poncirin	Flavonoid_179	Flavanones
180	Spiraeoside	Flavonoid_180	Flavonols
181	Demethyltexasin	Flavonoid_181	Isoflavanones
182	2'-Hydroxydaidzein	Flavonoid_182	Isoflavanones
183	2'-Hydroxygenistein	Flavonoid_183	Isoflavanones
184	Afzelechin	Flavonoid_184	Flavanols
185	3,7-Di-O-methylquercetin	Flavonoid_185	Flavonols
186	Prunetin	Flavonoid_186	Isoflavanones
187	Tricetin	Flavonoid_187	Flavones
188	Laricitrin	Flavonoid_188	Flavonols
189	Robinin	Flavonoid_189	Flavonols
190	7,4-Dihydroxyflavone	Flavonoid_190	Flavones
191	Naringenin chalcone	Flavonoid_191	Chalcones
192	Orientin	Flavonoid_192	Flavone glycosides
193	hydroxygenkwanin	Flavonoid_193	Flavones
194	Quercimeritrin	Flavonoid_194	Flavonols
195	(-)-Gallocatechin	Flavonoid_195	Flavanols
196	Pedalitin	Flavonoid_196	Flavones
197	Isorhamnetin 3-O-glucoside	Flavonoid_197	Flavonols
198	5,7-Dihydroxy-3,4,5-trimethoxyflavone	Flavonoid_198	Flavones
199	Persicogenin	Flavonoid_199	Flavanones
200	Benzylideneacetophenone	Flavonoid_200	Chalcones
201	Trilobatin	Flavonoid_201	Chalcones
202	Sieboldin	Flavonoid_202	Chalcones
203	Limocitrin	Flavonoid_203	Flavones
204	Linarin	Flavonoid_204	Flavones
