# Supplementary material for: (−)-Gallocatechin Gallate: A Novel Chemical Marker to Distinguish Triadica cochinchinensis Honey
Source: Foods. 2024 Jun 14;13(12):1879. doi: 10.3390/foods13121879 (PMC11203108; doi:10.3390/foods13121879)
Supplement: Supplementary file 1 [file foods-13-01879-s001.zip › Supplementary Table S2.pdf]

Table S2.

The specific flavonoid standards monitored in positive and negative ion modes.

No.	Abbreviations	Ion mode
1	Flavonoid_01	Negative
2	Flavonoid_02	Negative
3	Flavonoid_03	Positive
4	Flavonoid_04	Negative
5	Flavonoid_05	Positive
6	Flavonoid_06	Negative
7	Flavonoid_07	Positive
8	Flavonoid_08	Positive
9	Flavonoid_09	Negative
10	Flavonoid_10	Negative
11	Flavonoid_11	Negative
12	Flavonoid_12	Negative
13	Flavonoid_13	Negative
14	Flavonoid_14	Positive
15	Flavonoid_15	Negative
16	Flavonoid_16	Positive
17	Flavonoid_17	Negative
18	Flavonoid_18	Positive
19	Flavonoid_19	Negative
20	Flavonoid_20	Negative
21	Flavonoid_21	Positive
22	Flavonoid_22	Negative
23	Flavonoid_23	Negative
24	Flavonoid_24	Negative
25	Flavonoid_25	Negative
26	Flavonoid_26	Negative
27	Flavonoid_27	Positive
28	Flavonoid_28	Negative
29	Flavonoid_29	Negative
30	Flavonoid_30	Negative
31	Flavonoid_31	Negative
32	Flavonoid_32	Negative
33	Flavonoid_33	Negative
34	Flavonoid_34	Negative
35	Flavonoid_35	Negative
36	Flavonoid_36	Negative
37	Flavonoid_37	Negative
38	Flavonoid_38	Negative
39	Flavonoid_39	Positive
40	Flavonoid_40	Negative
41	Flavonoid_41	Negative

Continue for Table S2

No.	Abbreviations	Ion mode
42	Flavonoid_42	Negative
43	Flavonoid_43	Negative
44	Flavonoid_44	Negative
45	Flavonoid_45	Positive
46	Flavonoid_46	Negative
47	Flavonoid_47	Negative
48	Flavonoid_48	Negative
49	Flavonoid_49	Negative
50	Flavonoid_50	Negative
51	Flavonoid_51	Negative
52	Flavonoid_52	Negative
53	Flavonoid_53	Negative
54	Flavonoid_54	Negative
55	Flavonoid_55	Negative
56	Flavonoid_56	Negative
57	Flavonoid_57	Negative
58	Flavonoid_58	Negative
59	Flavonoid_59	Negative
60	Flavonoid_60	Negative
61	Flavonoid_61	Negative
62	Flavonoid_62	Negative
63	Flavonoid_63	Negative
64	Flavonoid_64	Negative
65	Flavonoid_65	Negative
66	Flavonoid_66	Negative
67	Flavonoid_67	Negative
68	Flavonoid_68	Negative
69	Flavonoid_69	Negative
70	Flavonoid_70	Negative
71	Flavonoid_71	Negative
72	Flavonoid_72	Negative
73	Flavonoid_73	Negative
74	Flavonoid_74	Negative
75	Flavonoid_75	Negative
76	Flavonoid_76	Negative
77	Flavonoid_77	Positive
78	Flavonoid_78	Negative
79	Flavonoid_79	Negative
80	Flavonoid_80	Negative
81	Flavonoid_81	Negative
82	Flavonoid_82	Negative
83	Flavonoid_83	Positive

Continue for Table S2

No.	Abbreviations	Ion mode
84	Flavonoid_84	Positive
85	Flavonoid_85	Positive
86	Flavonoid_86	Negative
87	Flavonoid_87	Positive
88	Flavonoid_88	Positive
89	Flavonoid_89	Negative
90	Flavonoid_90	Negative
91	Flavonoid_91	Negative
92	Flavonoid_92	Negative
93	Flavonoid_93	Negative
94	Flavonoid_94	Positive
95	Flavonoid_95	Negative
96	Flavonoid_96	Negative
97	Flavonoid_97	Negative
98	Flavonoid_98	Positive
99	Flavonoid_99	Negative
100	Flavonoid_100	Negative
101	Flavonoid_101	Negative
102	Flavonoid_102	Negative
103	Flavonoid_103	Positive
104	Flavonoid_104	Negative
105	Flavonoid_105	Positive
106	Flavonoid_106	Negative
107	Flavonoid_107	Negative
108	Flavonoid_108	Negative
109	Flavonoid_109	Positive
110	Flavonoid_110	Negative
111	Flavonoid_111	Positive
112	Flavonoid_112	Negative
113	Flavonoid_113	Negative
114	Flavonoid_114	Negative
115	Flavonoid_115	Negative
116	Flavonoid_116	Negative
117	Flavonoid_117	Negative
118	Flavonoid_118	Negative
119	Flavonoid_119	Negative
120	Flavonoid_120	Negative
121	Flavonoid_121	Negative
122	Flavonoid_122	Negative
123	Flavonoid_123	Negative
124	Flavonoid_124	Negative
125	Flavonoid_125	Negative

Continue for Table S2

No.	Abbreviations	Ion mode
126	Flavonoid_126	Negative
127	Flavonoid_127	Negative
128	Flavonoid_128	Positive
129	Flavonoid_129	Negative
130	Flavonoid_130	Negative
131	Flavonoid_131	Negative
132	Flavonoid_132	Negative
133	Flavonoid_133	Positive
134	Flavonoid_134	Negative
135	Flavonoid_135	Negative
136	Flavonoid_136	Negative
137	Flavonoid_137	Negative
138	Flavonoid_138	Negative
139	Flavonoid_139	Negative
140	Flavonoid_140	Negative
141	Flavonoid_141	Negative
142	Flavonoid_142	Positive
143	Flavonoid_143	Negative
144	Flavonoid_144	Positive
145	Flavonoid_145	Negative
146	Flavonoid_146	Negative
147	Flavonoid_147	Negative
148	Flavonoid_148	Negative
149	Flavonoid_149	Negative
150	Flavonoid_150	Negative
151	Flavonoid_151	Positive
152	Flavonoid_152	Negative
153	Flavonoid_153	Negative
154	Flavonoid_154	Negative
155	Flavonoid_155	Negative
156	Flavonoid_156	Negative
157	Flavonoid_157	Negative
158	Flavonoid_158	Negative
159	Flavonoid_159	Negative
160	Flavonoid_160	Negative
161	Flavonoid_161	Negative
162	Flavonoid_162	Positive
163	Flavonoid_163	Negative
164	Flavonoid_164	Positive
165	Flavonoid_165	Negative
166	Flavonoid_166	Negative
167	Flavonoid_167	Negative

Continue for Table S2

No.	Abbreviations	Ion mode
168	Flavonoid_168	Positive
169	Flavonoid_169	Negative
170	Flavonoid_170	Negative
171	Flavonoid_171	Negative
172	Flavonoid_172	Negative
173	Flavonoid_173	Positive
174	Flavonoid_174	Negative
175	Flavonoid_175	Negative
176	Flavonoid_176	Negative
177	Flavonoid_177	Negative
178	Flavonoid_178	Negative
179	Flavonoid_179	Negative
180	Flavonoid_180	Negative
181	Flavonoid_181	Negative
182	Flavonoid_182	Negative
183	Flavonoid_183	Negative
184	Flavonoid_184	Negative
185	Flavonoid_185	Negative
186	Flavonoid_186	Negative
187	Flavonoid_187	Negative
188	Flavonoid_188	Negative
189	Flavonoid_189	Negative
190	Flavonoid_190	Negative
191	Flavonoid_191	Negative
192	Flavonoid_192	Negative
193	Flavonoid_193	Positive
194	Flavonoid_194	Negative
195	Flavonoid_195	Negative
196	Flavonoid_196	Negative
197	Flavonoid_197	Negative
198	Flavonoid_198	Positive
199	Flavonoid_199	Negative
200	Flavonoid_200	Positive
201	Flavonoid_201	Negative
202	Flavonoid_202	Negative
203	Flavonoid_203	Negative
204	Flavonoid_204	Negative
