# Supplementary material for: (−)-Gallocatechin Gallate: A Novel Chemical Marker to Distinguish Triadica cochinchinensis Honey
Source: Foods. 2024 Jun 14;13(12):1879. doi: 10.3390/foods13121879 (PMC11203108; doi:10.3390/foods13121879)
Supplement: Supplementary file 1 [file foods-13-01879-s001.zip › Supplementary Table S3.pdf]

Table S3

Standard working curve information for 204 flavonoids.

No.	Abbreviations	RT (min)	Standard curve	Regression (R2)
1	Flavonoid_01	2.97	y = 3496.64251 x + 512.90946	0.99007
2	Flavonoid_02	2.86	y = 15566.53894 x - 7952.22892	0.99596
3	Flavonoid_03	9.09	y = 1.81793e5 x + 4.55831e4	0.99789
4	Flavonoid_04	4.96	y = 9620.15759 x - 4121.49541	0.99932
5	Flavonoid_05	8.10	y = 1.86812e5 x + 1.26563e6	0.99241
6	Flavonoid_06	5.17	y = 7.18068e4 x + 26486.33278	0.99664
7	Flavonoid_07	2.74	y = 258.20689 x - 454.01858	0.99460
8	Flavonoid_08	9.82	y = 6.81494e5 x + 4.37223e5	0.99306
9	Flavonoid_09	5.00	y = 9046.98507 x - 3748.22494	0.99223
10	Flavonoid_10	5.73	y = 21428.81692 x - 153.45325	0.99924
11	Flavonoid_11	2.06	y = 1656.79826 x + 55.31816	0.99895
12	Flavonoid_12	4.13	y = 3.51463e4 x - 193.91546	0.99849
13	Flavonoid_13	2.54	y = 21612.78102 x + 1593.03568	0.99559
14	Flavonoid_14	7.02	y = 4.05439e5 x + 3.01996e5	0.99463
15	Flavonoid_15	3.22	y = 4.66312e4 x - 5074.54552	0.99865
16	Flavonoid_16	9.63	y = 1.43238e5 x + 3.36773e4	0.99979
17	Flavonoid_17	8.20	y = 7.60344e4 x + 14249.96060	0.99959
18	Flavonoid_18	5.41	y = 19255.83059 x + 8918.37310	0.99913
19	Flavonoid_19	13.42	y = 2743.15245 x + 1412.01097	0.99792
20	Flavonoid_20	6.08	y = 19712.91851 x + 1238.15209	0.99845
21	Flavonoid_21	7.81	y = 3.67877e5 x + 1.53504e5	0.99611
22	Flavonoid_22	3.64	y = 5.41609e4 x - 25583.98227	0.99822
23	Flavonoid_23	4.24	y = 18188.43761 x - 8997.41754	0.99903
24	Flavonoid_24	6.85	y = 29093.86244 x + 4773.17867	0.99963
25	Flavonoid_25	3.18	y = 7764.49440 x - 536.53123	0.99897
26	Flavonoid_26	5.93	y = 1.06426e5 x + 13254.84795	0.99975
27	Flavonoid_27	8.27	y = 7831.44709 x - 8019.81928	0.99894
28	Flavonoid_28	2.85	y = 4213.01988 x + 1709.41040	0.99620
29	Flavonoid_29	5.59	y = 3.18632e4 x - 1500.00505	0.99924
30	Flavonoid_30	5.68	y = 4.69337e4 x + 9698.43490	0.99879
31	Flavonoid_31	3.41	y = 3.17601e4 x - 13073.30379	0.99622
32	Flavonoid_32	2.59	y = 4095.68295 x + 3459.58594	0.99648
33	Flavonoid_33	3.56	y = 5608.13139 x - 4688.87787	0.99904
34	Flavonoid_34	8.38	y = 3.71355e4 x + 18451.60567	0.99922
35	Flavonoid_35	3.11	y = 668.93192 x + 6033.72501	0.99919
36	Flavonoid_36	6.40	y = 5190.66292 x - 2131.67174	0.99433
37	Flavonoid_37	7.23	y = 14140.92564 x - 2421.02312	0.99836
38	Flavonoid_38	10.17	y = 15218.56178 x + 1937.59777	0.99923
39	Flavonoid_39	6.73	y = 2.08813e5 x + 5.87137e5	0.99440
40	Flavonoid_40	2.65	y = 4287.19487 x - 331.84649	0.99729
41	Flavonoid_41	5.24	y = 3.31398e4 x + 10695.98280	0.99941

Continue for Table S3

No.	Abbreviations	RT (min)	Standard curve	Regression (R2)
42	Flavonoid_42	2.92	y = 25483.97078 x - 11416.31051	0.99713
43	Flavonoid_43	5.10	y = 1552.77098 x + 952.12162	0.99882
44	Flavonoid_44	6.74	y = 26114.51304 x + 2963.74061	0.99979
45	Flavonoid_45	4.17	y = 209.89732 x + 2426.87207	0.99805
46	Flavonoid_46	6.49	y = 8.18200e4 x + 2998.92927	0.99990
47	Flavonoid_47	8.17	y = 4.02986e4 x + 17623.13545	0.99906
48	Flavonoid_48	2.97	y = 16247.22837 x - 1296.17060	0.99980
49	Flavonoid_49	3.53	y = 3023.03837 x + 5882.79644	0.99507
50	Flavonoid_50	4.19	y = 18298.50566 x + 3741.68481	0.99948
51	Flavonoid_51	2.38	y = 15023.45674 x + 2622.02085	0.99943
52	Flavonoid_52	2.78	y = 17028.06345 x - 2318.09435	0.99930
53	Flavonoid_53	8.46	y = 17251.46165 x + 462.60473	0.99851
54	Flavonoid_54	2.80	y = 4.28581e4 x - 649.13189	0.99994
55	Flavonoid_55	2.23	y = 1478.49444 x + 294.63848	0.99926
56	Flavonoid_56	3.13	y = 6293.82625 x - 22503.41482	0.99804
57	Flavonoid_57	2.72	y = 4.01514e4 x - 11812.44682	0.99699
58	Flavonoid_58	5.26	y = 3.43764e4 x + 1499.01561	0.99855
59	Flavonoid_59	5.08	y = 4918.80682 x + 2125.78818	0.99981
60	Flavonoid_60	10.95	y = 7.07044e4 x + 12165.52374	0.99860
61	Flavonoid_61	6.94	y = 6722.57196 x - 32.11654	0.99988
62	Flavonoid_62	9.79	y = 16841.90080 x - 616.90987	0.99970
63	Flavonoid_63	5.10	y = 5489.46620 x + 1972.90056	0.99989
64	Flavonoid_64	3.71	y = 4277.33303 x + 11537.65708	0.99542
65	Flavonoid_65	3.37	y = 8462.73189 x - 2785.66582	0.99181
66	Flavonoid_66	2.31	y = 3.74438e4 x + 10111.05700	0.99969
67	Flavonoid_67	3.50	y = 8525.55651 x + 28036.68406	0.99271
68	Flavonoid_68	2.30	y = 14312.70208 x - 487.94456	0.99977
69	Flavonoid_69	2.88	y = 24506.14522 x + 4609.24416	0.99897
70	Flavonoid_70	7.82	y = 15622.50855 x + 4433.00848	0.99851
71	Flavonoid_71	7.21	y = 1762.75106 x + 444.83892	0.99804
72	Flavonoid_72	4.94	y = 12713.88532 x - 756.19109	0.99986
73	Flavonoid_73	2.98	y = 3388.44047 x + 3006.49162	0.99846
74	Flavonoid_74	2.47	y = 4738.76317 x - 2050.63501	0.99502
75	Flavonoid_75	8.05	y = 14435.73063 x + 3782.69761	0.99939
76	Flavonoid_76	7.00	y = 3.94561e4 x + 9731.33545	0.99947
77	Flavonoid_77	3.73	y = 72.35034 x - 1078.26608	0.99338
78	Flavonoid_78	2.72	y = 17532.89145 x - 1799.49114	0.99780
79	Flavonoid_79	2.80	y = 10061.74193 x + 923.32920	0.99535
80	Flavonoid_80	2.54	y = 1010.75160 x - 2834.40065	0.99940
81	Flavonoid_81	3.96	y = 3.28620e4 x + 8.30138e4	0.99729
82	Flavonoid_82	6.97	y = 3.79017e4 x + 5935.52047	0.99976
83	Flavonoid_83	3.87	y = 87.63503 x + 211.78651	0.99174

Continue for Table S3

No.	Abbreviations	RT (min)	Standard curve	Regression (R2)
84	Flavonoid_84	2.00	y = 3591.02493 x + 4181.99966	0.99879
85	Flavonoid_85	3.88	y = 147.05366 x + 5794.84459	0.99421
86	Flavonoid_86	3.07	y = 4.40745e4 x - 17715.64103	0.99611
87	Flavonoid_87	9.60	y = 3.96674e4 x + 22551.23046	0.99916
88	Flavonoid_88	8.56	y = 1.43485e5 x + 4.09148e4	0.99958
89	Flavonoid_89	4.69	y = 3234.29590 x - 321.54766	0.99921
90	Flavonoid_90	2.86	y = 27001.82769 x - 157.29945	0.99799
91	Flavonoid_91	10.98	y = 27014.89076 x + 4740.02193	0.99883
92	Flavonoid_92	3.47	y = 16340.26158 x - 3.95252e4	0.99813
93	Flavonoid_93	7.62	y = 5.97713e4 x + 9815.26867	0.99903
94	Flavonoid_94	3.79	y = 12.89204 x - 134.02025	0.99025
95	Flavonoid_95	4.28	y = 4.64528e4 x - 56.06262	0.99986
96	Flavonoid_96	2.78	y = 142.94362 x + 2283.45075	0.99286
97	Flavonoid_97	9.56	y = 24333.33376 x + 2883.87911	0.99915
98	Flavonoid_98	2.83	y = 38.57300 x + 1772.72199	0.99491
99	Flavonoid_99	7.02	y = 5.55672e4 x + 3.03102e4	0.99779
100	Flavonoid_100	8.54	y = 4.06462e4 x + 7169.35674	0.99914
101	Flavonoid_101	3.12	y = 14697.51797 x - 2326.09035	0.99908
102	Flavonoid_102	3.67	y = 177.17537 x + 49.48761	0.99772
103	Flavonoid_103	7.07	y = 4.81070e5 x + 2.06068e6	0.99505
104	Flavonoid_104	8.32	y = 28107.08811 x + 4828.16421	0.99958
105	Flavonoid_105	10.74	y = 4.31727e5 x + 1.62499e5	0.99770
106	Flavonoid_106	2.60	y = 7746.69288 x - 1832.87360	0.99957
107	Flavonoid_107	6.89	y = 2745.17009 x + 349.62545	0.99948
108	Flavonoid_108	7.13	y = 4479.68964 x + 1063.09169	0.99864
109	Flavonoid_109	9.41	y = 9848.84667 x + 29416.79056	0.99644
110	Flavonoid_110	7.27	y = 9.92569e4 x + 6.65409e4	0.99982
111	Flavonoid_111	6.70	y = 4.60853e5 x + 9.03464e4	0.99771
112	Flavonoid_112	4.89	y = 6261.73019 x + 10223.42241	0.99744
113	Flavonoid_113	2.64	y = 553.45382 x - 3904.94602	0.99182
114	Flavonoid_114	3.29	y = 5187.88386 x - 62.56165	0.99864
115	Flavonoid_115	3.12	y = 17884.29517 x - 7175.78110	0.99512
116	Flavonoid_116	11.18	y = 1.96199e5 x + 5.74236e4	0.99924
117	Flavonoid_117	3.25	y = 6253.96257 x - 1655.74008	0.99929
118	Flavonoid_118	2.94	y = 24285.22180 x - 5030.49433	0.99884
119	Flavonoid_119	3.07	y = 28244.96221 x - 5340.27143	0.99963
120	Flavonoid_120	9.85	y = 3.37491e4 x - 854.95011	0.99958
121	Flavonoid_121	8.04	y = 3.11656e4 x + 9026.94990	0.99793
122	Flavonoid_122	2.67	y = 2680.27398 x + 1584.75257	0.99873
123	Flavonoid_123	2.85	y = 17627.91118 x - 9.60745e4	0.99000
124	Flavonoid_124	3.69	y = 5751.25973 x - 917.63861	0.99971
125	Flavonoid_125	5.64	y = 4.16798e4 x + 6499.97194	0.99932

Continue for Table S3

No.	Abbreviations	RT (min)	Standard curve	Regression (R2)
126	Flavonoid_126	4.15	y = 4550.56713 x - 1734.30662	0.99933
127	Flavonoid_127	3.98	y = 0.03415 x + 0.01082	0.99962
128	Flavonoid_128	9.30	y = 7.19107e4 x + 3.02137e4	0.99918
129	Flavonoid_129	8.75	y = 5.30150e4 x + 4159.39120	0.99959
130	Flavonoid_130	9.45	y = 4.58462e4 x + 6754.16247	0.99968
131	Flavonoid_131	7.28	y = 5.08594e4 x + 9613.84562	0.99929
132	Flavonoid_132	5.21	y = 5585.20817 x + 2026.04124	0.99732
133	Flavonoid_133	11.97	y = 1.97723e5 x + 2.09279e5	0.99960
134	Flavonoid_134	7.31	y = 13086.43890 x + 7698.20118	0.99944
135	Flavonoid_135	3.88	y = 221.96227 x - 5295.08012	0.99470
136	Flavonoid_136	4.75	y = 14406.24304 x + 1272.72746	0.99992
137	Flavonoid_137	3.97	y = 5.07516e4 x - 5741.66653	0.99954
138	Flavonoid_138	2.49	y = 3.01299e4 x - 9294.51881	0.99932
139	Flavonoid_139	3.62	y = 10534.03280 x - 2360.14674	0.99893
140	Flavonoid_140	6.82	y = 26209.34480 x + 8287.43209	0.99734
141	Flavonoid_141	4.45	y = 13145.87538 x - 2461.01797	0.99910
142	Flavonoid_142	10.31	y = 3.76660e4 x + 18470.16589	0.99921
143	Flavonoid_143	5.29	y = 3.31399e4 x + 3067.26388	0.99978
144	Flavonoid_144	6.62	y = 5.28585e4 x + 18532.57539	0.99870
145	Flavonoid_145	2.28	y = 568.70457 x + 34.08694	0.99888
146	Flavonoid_146	4.08	y = 16.67465 x - 21.50348	0.99932
147	Flavonoid_147	2.46	y = 2459.52067 x - 1253.04103	0.99937
148	Flavonoid_148	7.10	y = 8.61118e4 x + 18162.33444	0.99929
149	Flavonoid_149	6.58	y = 13943.26561 x + 438.99537	0.99994
150	Flavonoid_150	2.92	y = 4967.16905 x - 3246.08227	0.99643
151	Flavonoid_151	6.29	y = 7.55182e5 x + 8.67117e4	0.99941
152	Flavonoid_152	3.62	y = 202.27683 x + 243.75877	0.99171
153	Flavonoid_153	2.63	y = 400.66950 x + 176.41939	0.99492
154	Flavonoid_154	2.79	y = 5.69540e4 x - 12486.38150	0.99727
155	Flavonoid_155	2.67	y = 2803.40938 x + 330.40111	0.99967
156	Flavonoid_156	4.25	y = 1184.12184 x - 313.90256	0.99505
157	Flavonoid_157	2.38	y = 24759.27226 x - 442.55661	0.99954
158	Flavonoid_158	7.06	y = 12920.81549 x + 271.67282	0.99987
159	Flavonoid_159	10.49	y = 11171.78777 x + 3435.89476	0.99851
160	Flavonoid_160	3.14	y = 25214.69355 x + 8643.20875	0.99960
161	Flavonoid_161	3.05	y = 3.70536e4 x - 4780.78550	0.99920
162	Flavonoid_162	9.58	y = 1.80247e5 x + 4.55074e4	0.99869
163	Flavonoid_163	6.87	y = 6094.88840 x + 2582.36375	0.99949
164	Flavonoid_164	6.42	y = 1.72394e5 x + 7.51976e5	0.99278
165	Flavonoid_165	3.21	y = 4.64092e4 x + 3024.27303	0.99942
166	Flavonoid_166	7.13	y = 5.17847e4 x + 13639.12792	0.99965
167	Flavonoid_167	3.03	y = 2756.47241 x + 1589.63213	0.99470

Continue for Table S3

No.	Abbreviations	RT (min)	Standard curve	Regression (R2)
168	Flavonoid_168	5.96	y = 9.82381e5 x + 3.36929e5	0.99887
169	Flavonoid_169	2.70	y = 18533.08138 x - 1994.84339	0.99962
170	Flavonoid_170	8.92	y = 9817.40549 x + 986.47041	0.99924
171	Flavonoid_171	5.84	y = 16500.93425 x + 8401.62724	0.99918
172	Flavonoid_172	2.78	y = 10383.84237 x + 1676.58393	0.99892
173	Flavonoid_173	8.54	y = 1.87218e5 x + 5.78068e4	0.99795
174	Flavonoid_174	8.76	y = 3.13112e4 x + 4211.03658	0.99969
175	Flavonoid_175	3.42	y = 3.35828e4 x + 3299.04594	0.99788
176	Flavonoid_176	3.20	y = 2477.66244 x + 9678.26546	0.99897
177	Flavonoid_177	4.87	y = 4.77779e4 x - 9920.06526	0.99930
178	Flavonoid_178	2.82	y = 2884.40000 x - 13159.88696	0.99424
179	Flavonoid_179	4.06	y = 5.52843e4 x - 16385.37072	0.99861
180	Flavonoid_180	3.16	y = 3.22786e4 x - 6831.95104	0.99765
181	Flavonoid_181	3.46	y = 4471.47986 x - 3738.10768	0.99815
182	Flavonoid_182	3.52	y = 22537.32223 x + 2161.08798	0.99909
183	Flavonoid_183	4.04	y = 27073.69708 x + 7065.48965	0.99891
184	Flavonoid_184	2.58	y = 3410.56945 x - 1499.56366	0.99867
185	Flavonoid_185	6.46	y = 1.35334e5 x + 5.77167e4	0.99895
186	Flavonoid_186	7.31	y = 13065.82138 x + 2684.89791	0.99849
187	Flavonoid_187	3.52	y = 3.59756e4 x - 8072.66137	0.99900
188	Flavonoid_188	4.25	y = 18391.03874 x + 3925.67337	0.99842
189	Flavonoid_189	2.49	y = 13417.33235 x - 4.65892e4	0.99504
190	Flavonoid_190	3.73	y = 12762.39776 x + 9359.78856	0.99850
191	Flavonoid_191	4.94	y = 15185.77868 x + 11139.29097	0.99582
192	Flavonoid_192	2.61	y = 21001.13494 x - 6.12842e4	0.99438
193	Flavonoid_193	6.04	y = 29989.41428 x + 10415.52516	0.99949
194	Flavonoid_194	2.82	y = 1262.66827 x + 3665.56751	0.99603
195	Flavonoid_195	1.55	y = 4516.03332 x - 759.39135	0.99918
196	Flavonoid_196	3.69	y = 5.69893e4 x + 2839.39269	0.99953
197	Flavonoid_197	3.10	y = 29069.36487 x - 4466.42695	0.99574
198	Flavonoid_198	6.74	y = 6.60145e4 x + 20497.33408	0.99762
199	Flavonoid_199	7.29	y = 812.04703 x - 2050.19878	0.99902
200	Flavonoid_200	9.36	y = 8.72597e4 x + 16781.76422	0.99990
201	Flavonoid_201	3.59	y = 28836.75590 x - 6991.59931	0.99802
202	Flavonoid_202	3.10	y = 5261.10220 x - 4988.75140	0.99246
203	Flavonoid_203	3.79	y = 2306.05066 x - 3624.52031	0.99957
204	Flavonoid_204	3.77	y = 35.26563 x - 581.20672	0.99245
